# Matrix metalloproteinase-2 and -9 and tissue inhibitor of metalloproteinase-1 and -2 in sera and urine of patients with renal carcinoma

**DOI:** 10.3892/ol.2013.1755

**Published:** 2013-12-11

**Authors:** ANGELINA DI CARLO

**Affiliations:** Department of Medico-Surgical Sciences and Biotechnologies, ‘Sapienza’ University of Rome, Latina I-04100, Italy

**Keywords:** kidney carcinoma, matrix metalloproteinase-2, matrix metalloproteinase-9, tissue inhibitor of metalloproteinase-1, tissue inhibitor of metalloproteinase-2, sera, urine

## Abstract

The matrix metalloproteinase (MMP) family has been shown to play a critical role in tissue remodeling and tumor infiltration. Their activity is normally strictly controlled by tissue inhibitors of metalloproteinases (TIMPs). However, TIMPs act indirectly through modulation of protease activity or directly through cell surface receptors to direct cell fate. These molecules have been proposed as markers of malignant cancer. Previous studies on MMP and TIMP expression in kidney carcinoma have been limited and have reported variable observations. The current study measured the content of MMP-2 and -9 and TIMP-1 and -2 in the sera and urine of patients with kidney carcinoma by enzyme-linked immunosorbent assay. Of these patients, 16 exhibited clear cell renal cell carcinoma (ccRCC) and 4 exhibited oncocytoma. Sera and urine samples of 53 healthy subjects were used as controls. In the sera of the control group, MMP-2 and TIMP-2 were detectable in all samples, while MMP-9 and TIMP-1 were below the sensitivity of the assay. In the pathological specimens, the mean serum values of MMP-2 and TIMP-1 and -2 were similar in the ccRCC and oncocytoma patients, whereas the value for MMP-9 was 2-fold higher in the ccRCC patients compared with the oncocytoma patients. With regard to the urine specimens, all four molecules were undetectable in the normal healthy samples and in a few pathological samples. The mean values for MMP-2 and -9 and TIMP-2 in the positive urine specimens were similar in the ccRCC and oncocytoma patients, whereas the mean value of TIMP-1 was higher in the ccRCC patients compared with that of the oncocytoma patients. The mean urinary levels of the four molecules were less than those of the sera. Statistical analysis of the data did not reveal any correlation between the tumor grades and expression levels of the molecules examined.

## Introduction

Kidney tumors are the third most common malignancy of the urinary tract following prostate and bladder cancer (1). Kidney tumors have no apparent symptoms and are frequently fatal, and the majority are incidentally found during routine abdominal imaging performed for unrelated reasons. In addition, kidney biopsy is an invasive technique that may result in complications and is unlikely to provide accurate diagnosis in certain situations (2). Pre-existing screening tests are available for organ-specific tumors, including prostate-specific antigen and digital rectal examination for prostate cancer, carcinoembryonic antigen, colonoscopy and testing for fecal occult blood for colon cancer, and mammography and breast examinations for breast cancer, however, there are no diagnostic modalities for the early detection of renal cancer and no methods for the surveillance of its recurrence. Thus, there is great interest in identifying soluble biomarkers that may improve this situation.

Matrix metalloproteinases (MMPs) form a family of >25 endopeptidases. MMPs may be described as multifunctional enzymes capable of cleaving the basal membrane, extracellular matrix components (such as fibrillar and non-fibrillar collagen, proteoglycans, glycoproteins and denatured collagen), growth factors, cytokines and cell surface-associated adhesion and signaling receptors. Therefore, MMPs enhance tumor growth and tumorigenicity. In particular, MMP-2 and -9, also known as gelatinase A (72 kDa) and B (92 kDa), respectively, are most often associated with the malignant phenotype of tumor cells. Growing amounts of data indicate that circulating MMP-9 and/or MMP-2 levels may be valuable in assessing prognosis or diagnosing a relapse during follow-up (3–5). Due to their high degradation activity and potentially disastrous effect on the cell microenvironment, cellular MMPs are expressed in small amounts and their cellular localization and activity are tightly controlled. Normally, MMPs are inhibited by tissue inhibitors of metalloproteinases (TIMPs), and the MMP/TIMP balance is considered to be a major factor in the regulation of the net proteolytic activity of the individual MMPs. In humans, four individual species of TIMPs are known (TIMP-1, -2, -3 and -4). Of these, the C-terminal domain of TIMP-1 and -2 bind the to the hemopexin domain of the proenzymes of MMP-9 and -2, respectively (6,7). TIMP expression is regulated during development and tissue remodeling and under pathological conditions associated with unbalanced MMP activities. Changes in TIMP levels are considered to be important since they directly affect the levels of MMP activity. Additionally, TIMP-2 is unique in that, as well as inhibiting the activity of MMP, it selectively interacts with membrane type-1 MMP (MT1-MMP) to facilitate the cell surface activation of the precursor of MMP-2 (pro-MMP-2). Thus, TIMP-2 functions to inhibit MMP activity and promote the cell surface activation of pro-MMP-2 by MT1-MMP interaction. In addition to metalloproteinase-inhibiting activities, TIMPs exhibit other biological functions and have been implicated in the direct regulation of the growth and apoptosis of cells (8–10).

Changes in MMPs and their inhibitors on the cellular level may be reflected in body fluids. This is likely to allow determination of MMPs and TIMPs in blood and/or urine as a simple non-invasive tool for cancer diagnosis and monitoring.

In the present study, the serum and urinary levels of MMP-2 and -9 and TIMP-1 and -2 were measured in patients with oncocytoma or clear cell renal cell carcinoma (ccRCC) in order to verify whether these molecules may offer a potential non-invasive biomarker to provide useful clinical information for kidney carcinoma.

## Materials and methods

### Patients

Peripheral venous blood and first morning urine samples were collected from 20 selected patients prior to surgical or other therapeutic intervention. Tumor specimens were obtained from patients who had undergone surgical procedure. Standard clinical laboratory criteria and histopathological observations were used to diagnose and confirm the tumor type. The patient ages ranged between 40 and 73 years (mean, 59.2±9.7 years), and in total, there were 9 females and 11 males. The tumors were classified by grade and stage according to the pTNM classification (11). All patients provided written informed consent and the study was approved by the local ethics committee. A total of 53 normal healthy volunteers with no concomitant illness were used as controls. The age of the healthy volunteers ranged between 30 and 70 years (mean, 42±8 years) and there were 30 females and 23 males. These volunteers provided verbal permission. The subjects in the controls exhibited no signs of infection, gastrointestinal hepatic or renal disease, tumors or immunological disease. The basic laboratory parameter values of these participants were within the reference limits.

### Serum

Native serum was prepared using plastic tubes without coagulation accelerators, to prevent the release of gelatinase during platelet activation. The tubes were centrifuged at 1,600 × g for 10 min, 30 min after blood collection. The sera were aliquoted and stored at −20°C prior to use.

### Urine sample preparation

The Multistix Combur test (Roche Diagnostic GmbH, Mannheim, Germany) was used to examine the urine samples prior to analysis. The urine samples that tested positive for leukocytes were excluded due to confounding leukocytic gelatinases. Microscopic hematuria, which was present in the majority of cancer samples, was not quantified, however, macroscopic hematuric samples were excluded. Immediately after collection, the samples were frozen and stored at −20°C prior to being assayed. For this, the samples were thawed and a 15-ml aliquot of each sample was centrifuged at 1,000 × g for 10 min at 4°C. Supernatant was collected and used to determine the MMP-2 and -9 and TIMP-1 and -2 levels by immunoassay.

### Measurement of MMP-2 and -9 and TIMP-1 and -2 levels

MMP-2 and -9 and TIMP-1 and -2 levels were detected by enzyme-linked immunosorbent assay (ELISA) using commercial kits obtained from GE Healthcare (Amersham, UK). These assays are based on a two-site sandwich format using two antibodies directed against various epitopes of the molecule. The assay for MMP-2 recognizes the pro-MMP-2, i.e., free pro-MMP-2 and that complexed with TIMP-2, but not the active form of MMP-2. The assay for MMP-9 recognizes the precursor of MMP-9 (pro-MMP-9), i.e., free pro-MMP-9 and that complexed with TIMP-1. The assay for TIMP-1 recognizes free TIMP-1 and that complexed with MMPs. The assay for TIMP-2 recognizes free TIMP-2 and that complexed with the active form of MMPs. All analyses were performed according to the manufacturer’s instructions.

### Statistical analysis

All statistical analyses were performed using the statistical computing environment R software (version 2.12.1; R Foundation for Statistical Computing, Vienna, Austria). Data are presented as the mean ± standard deviation (SD). Fisher’s exact test was performed and P<0.05 was considered to indicate a statistically significant difference.

## Results

### Patients

A total of 20 patients with kidney disease were evaluated over a 1-year period. Of these patients, 16 exhibited ccRCC and 4 exhibited oncocytoma. A venous blood sample was collected from each of the patients, and for all of the patients with oncocytoma and for nine of the patients with ccRCC, first morning urine samples were obtained. All four molecules, including MMP-2 and -9 and their inhibitors TIMP-2 and -1, were measured in the serum and urine samples. The levels of these molecules were also measured in the sera and urine of 53 healthy subjects, who were considered to be the control group, as normal values for these molecules were unavailable.

### Serum samples

**Figure 1:**
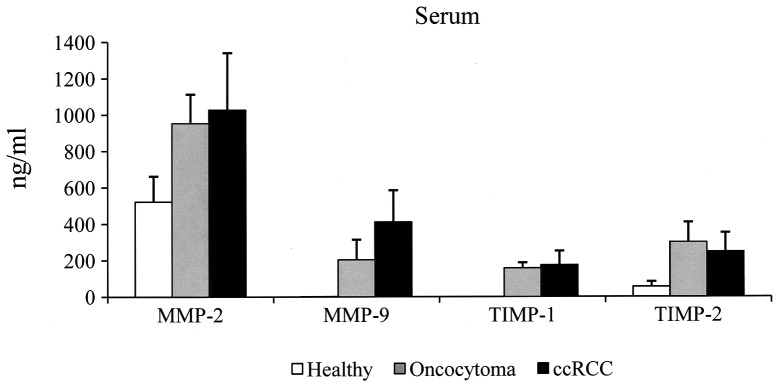
Data are presented as the mean ± SD of serum MMP-2 and -9 and TIMP-1 and -2 expression levels. MMP, matrix metalloproteinase; TIMP, tissue inhibitor of metalloproteinase; ccRCC, clear cell renal carcinoma; SD, standard deviation.

In the sera of the control group, MMP-2 was detected in all the samples, with a value ranging between 475 and 798 ng/ml (mean, 522±140 ng/ml). MMP-9 and TIMP-1 were undetectable, being at or below the sensitivity of the assay. TIMP-2 was detected in all the specimens and ranged between 33 and 118 ng/ml (mean, 55±28 ng/ml). The cut-off value was established by calculating the mean ± 2SD. The cut-off values of 802 ng/ml for MMP-2 and 111 ng/ml for TIMP-2 were used. Samples with a value higher than that of the cut-off were considered positive. The results obtained from the sera of the patients with oncocytoma and ccRCC are shown in Tables I and II, respectively. In the patients with oncocytoma, the MMP-2 values were positive in 3/4 (75%) of the specimens analyzed (range, 750–1,120 ng/ml; mean ± SD, 953±160 ng/ml), while in the patients with ccRCC, the values were positive in 12/16 (75%) of specimens (range, 697–1,949; mean ± SD, 1,027±314 ng/ml). MMP-9 was detected in all the specimens analyzed, with mean values of 203±111 ng/ml (range, 82–327 ng/ml) and 411±174 ng/ml (range, 168–730 ng/ml) observed in the oncocytoma and ccRCC patients, respectively. TIMP-1 was detected in all the specimens analyzed; with a mean value of 157±31 ng/ml (range, 120–186 ng/ml) in the oncocytoma patients and 174±78 (range, 63–429 ng/ml) in the ccRCC patients. Since serum MMP-9 and TIMP-1 were undetectable in all the healthy subjects, all pathological specimens were considered positive, as all samples possessed serum MMP-9 and TIMP-1 values higher than the sensitivity of the assay (assay sensitivity was calculated as 2SDs above the zero dose binding of 80 determinations, and was 0.8 ng/ml for MMP-9 and 1.51 ng/ml for TIMP-1). TIMP-2 was positive in all (100%) the individuals with oncocytoma (range, 184–445 ng/ml; mean ± SD, 300±110 ng/ml) and in 15/16 (94%) of the ccRCC patients (range, 52–452 ng/ml; mean ± SD, 248±105 ng/ml). Considering the average value of each molecule, the MMP-2 values were observed to be similar in the oncocytoma and ccRCC patients, however, the mean value was ~2-fold higher in the sera from the kidney disease patients compared with that of the control group. In addition, the serum MMP-9 level was 2-fold higher in the patients with ccRCC compared with those with oncocytoma (Fig. 1). We observed that TIMP-1 and -2 values were similar in oncocytoma and ccRCC individuals. In addition, serum TIMP-2 levels were ~5-fold higher in kidney disease compared with the healthy specimens (Fig. 1).

### Urine samples

**Figure 2:**
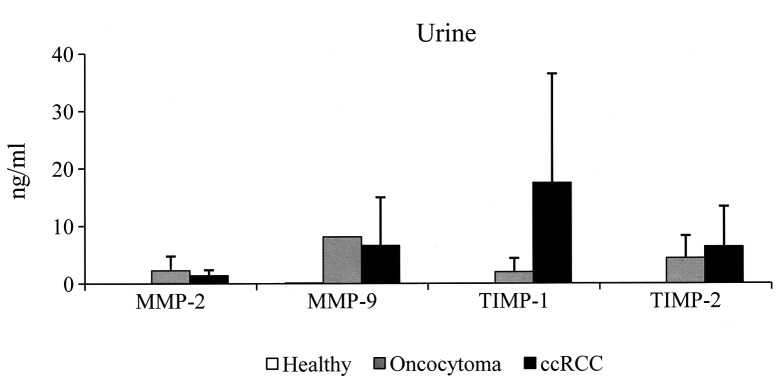
Data are presented as the mean ± SD of urinary MMP-2 and -9 and TIMP-1 and -2 expression levels. MMP, matrix metalloproteinase; TIMP, tissue inhibitor of metalloproteinase; ccRCC, clear cell renal carcinoma; SD, standard deviation.

With regard to the urine specimens, since the four molecules were undetectable in all the urine samples of the control group, no cut-off values were established. In Tables III and IV, the results obtained in the urine from patients with oncocytoma and ccRCC, respectively, are shown. In particular, urinary MMP-2 was detected in two (50%) oncocytoma specimens, with values of 0.58 and 4.11 ng/ml, respectively, and in 8/9 (89%) ccRCC samples (range, 0.72–3.41; mean ± SD, 1.4±1.0), whereas urinary MMP-9 was detected in only one (25%) oncocytoma specimen, with a value of 8.13 ng/ml, and in 6/9 (67%) of ccRCC patients (range, 0.55–22.8 ng/ml). TIMP-1 was detected in two (50%) oncocytoma individuals and in 8/9 (89%) of the ccRCC patients (range, 0.17–55 ng/ml). Finally, TIMP-2 was detected in 3/4 (75%) of the oncocytoma patients and in 7/9 (78%) of the ccRCC patients. The mean values of MMP-2 and -9 and TIMP-1 and -2 of the positive urine specimens are shown in Fig. 2. It is evident that the urinary levels were less than those of the sera. In addition, the mean level of urinary TIMP-1 was higher in the ccRCC patients compared with the oncocytoma patients.

## Discussion

To date, no reliable, non-invasive tumor markers for renal cell carcinoma have been described. Among the current tumor biomarkers, MMP members and their inhibitors (TIMPs) have the potential to represent candidates to improve diagnosis and follow-up surveillance. In humans, MMP expression has previously been reported to be increased in the majority of malignancies (3,4). TIMPs are multifunctional and act indirectly through modulation of protease activity or directly through cell surface receptors to direct cell fate (10). Tissue destruction in malignancies correlates with an imbalance of MMPs over TIMPs. An aberrant expression of TIMPs has been postulated to present an important modulatory and prognostic factor in the invasive capacity of specific tumors. Therefore, an imbalance between the expression, activation and presentation of MMP-2 and -9 and their associated TIMPs may have a role in the invasive phenotype. Previously, MMP-2 and -9 and TIMP-1 and -2 have been investigated, using diverse techniques, in body fluids and tissues with variable results (12–16). Tissue MMP-2 and -9 and TIMP-1 and -2 were found to be overexpressed in tumors and more frequently in non-ccRCC (13,14). In particular, using immunohistochemistry, Kallakury *et al* (14) reported that the increased expression levels of MMP-2 and -9 and TIMP-1 and -2 individually correlate with histological tumor types, with a vast majority of papillary and sarcomatoid RCCs expressing these proteins as compared with clear cell tumors. Furthermore, the increased expression of the four molecules was found to correlate with poor prognostic variables, including shortened patient survival (14). Using radioactive-labeled riboprobe *in situ* hybridization and immunohistochemistry, Bhuvarahamurthy *et al* analyzed the formalin-fixed, paraffin-embedded tumor samples of 10 patients and found the pronounced expression of MMP-2 and -9 in RCC at the mRNA and protein levels. In addition, the expression of TIMP-1 and -2 appeared to be relevant in RCC (15). However, although these studies on tissue markers are highly promising, there are certain limitations. Immunochemistry is semi-quantitative and highly dependent on a range variables, including choice of antibody, antibody concentration, fixation techniques, variability in the interpretation and stratification criteria and inconsistency in specimen handling and technical procedures. Using an RT-PCR technique, Kugler *et al* analyzed MMP-2 and -9 and TIMP-1 and -2 in 17 RCC patients and demonstrated a marked correlation between increased gene expression and tumor stage and aggressiveness (16). Kamiya *et al* found that the lytic activity is higher at the peripheries of tumors in inflammatory sites, as observed by *in situ* zymography (17). In addition, in 36 RCC patients, Lein *et al* evaluated the content of the four molecules using various techniques (RT-PCR, zymography, immunohistochemistry and ELISA). The study found that in the tumor tissues, MMP-9 and TIMP-1 were significantly higher than in the normal counterparts. The level of MMP-2 did not differ between the tumor and normal counterparts, and the measurement of the TIMP-2 values was not possible (18). With regard to peripheral blood, Lein *et al* found that the plasma MMP-9 levels were significantly higher in RCC patients than in healthy controls, whereas MMP-2 and TIMP-2 concentrations were higher in the healthy controls and the TIMP-1 concentrations were not different. In particular, the study reported that plasma MMP-9 showed a sensitivity of only 36% in detecting RCC, and no correlation was found with tumor type, grade or stage (18). Using a zymography technique, our previous study showed that MMP-9 is enhanced in the sera from ccRCC patients compared with that from oncocytoma patients, and that the most abundant lytic activity was at 92 kDa (MMP-9), whereas MMP-2 was present in reduced quantities (12). The results of the ELISA in the present study showed that MMP-2 and -9 and TIMP-1 and -2 were present in the sera from all kidney disease patients analyzed. The mean values of MMP-2 and TIMP-1 were similar in the ccRCC and oncocytoma patients, whereas the mean values of MMP-9 were higher in the ccRCC patients compared with those of oncocytoma patients. Therefore, according to Lein *et al,* the results of the current study support the hypothesis for the significance of MMP-9 in renal cancer, whereas MMP-2 does not appear to be important. However, a broad overlap of the results was identified, and no correlation was observed among the type of carcinoma, pathological TNM stage or histological grading.

The ability to follow localized tumors or monitor drug-based therapy results by a simple analysis of tumor-specific markers in the easily available excretory product of the kidney is desirable. However, to the best of our knowledge, insufficient literature exists concerning urine markers for RCC. In the urine samples of the present study, MMPs and TIMPs were detectable only in certain pathological specimens, whereas they were undetectable in the control group. No correlation was identified between the urinary levels of the four molecules analyzed and the clinical pathological observations. The results are consistent with the results of the study by Cannon *et al* (19), but contradict the results of the study by Sherief *et al* (20).

To date, despite tissue evidence, the analysis of serum and urine MMP and TIMP levels appears to be an inadequate test to identify kidney cancer. This conclusion may not be transferable to the general population, however, due to the small number of patients included in the studies. Therefore, further evaluation is required. Future investigation involving a larger cohort of patients may clarify whether MMP-2 and -9 and TIMP-1 and -2 are useful biomarkers for ccRCC.

## Figures and Tables

**Table I:** Serum MMP and TIMP content in oncocytoma patients.

Case	Age, years	Gender	Stage	Grade	MMP-2, ng/ml	MMP-9, ng/ml	TIMP-1, ng/ml	TIMP-2, ng/ml
1	42	F	T1N0M0	G1	1120	259	120	184
2	66	M	T1N0M0	G1	1030	142	177	310
3	59	F	T2N0M0	G1	750	82	186	261
4	59	F	T1N0M0	G1	910	327	143	445

MMP, matrix metalloproteinase; TIMP, tissue inhibitor of metalloproteinase.

**Table II:** Serum MMP and TIMP content in ccRCC patients.

Case	Age, years	Gender	Stage	Grade	MMP-2, ng/ml	MMP-9, ng/ml	TIMP-1, ng/ml	TIMP-2, ng/ml
5	69	M	T1N0M0	G1	1423	168	206	292
6	54	M	T1N0M0	G1	710	355	103	275
7	53	M	T1N0M0	G2	1175	173	226	123
8	60	F	T1N0M0	G2	697	609	125	52
9	51	F	T1N0M0	G2	1949	356	119	184
10	63	F	T1N0M0	G2	880	428	151	293
11	63	M	T2N0M0	G2	945	312	262	268
12	60	F	T2N0M0	G2	795	575	63	269
13	40	M	T2N0M0	G3	965	291	429	349
14	73	M	T2N0M0	G3	805	202	210	318
15	70	M	T2N0M0	G3	1060	497	150	326
16	61	M	T2N0M0	G3	1140	681	119	165
17	73	F	T2N0M0	G3	1030	730	82	124
18	43	M	T2N0M0	G3	1120	499	161	145
19	67	M	T3N0M1	G3	995	309	207	335
20	57	F	T3bN0M1	G3	745	393	172	452

MMP, matrix metalloproteinase; TIMP, tissue inhibitor of metalloproteinase; ccRCC, clear cell renal carcinoma.

**Table III:** Urine MMP and TIMP content in oncocytoma patients.

Case	Age, years	Gender	Stage	Grade	MMP-2, ng/ml	MMP-9, ng/ml	TIMP-1, ng/ml	TIMP-2, ng/ml
1	42	F	T1N0M0	G1	0.58	8.13	0.30	N.D.
2	66	M	T1N0M0	G1	4.11	N.D.	N.D.	4.14
3	59	F	T2N0M0	G1	N.D.	N.D.	3.77	8.51
4	59	F	T1N0M0	G1	N.D.	N.D.	N.D.	0.65

MMP, matrix metalloproteinase; TIMP, tissue inhibitor of metalloproteinase; N.D., none detectable.

**Table IV:** Urine MMP and TIMP content in ccRCC patients.

Case	Age, years	Gender	Stage	Grade	MMP-2, ng/ml	MMP-9, ng/ml	TIMP-1, ng/ml	TIMP-2, ng/ml
5	69	M	T1N0M0	G1	2.08	6.93	27.80	3.92
6	54	M	T1N0M0	G1	0.77	N.D.	N.D.	4.57
7	53	M	T1N0M0	G2	3.41	0.55	55.00	21.10
9	51	F	T1N0M0	G2	1.01	N.D.	1.70	1.84
13	40	M	T2N0M0	G3	0.72	1.68	0.51	6.70
14	73	M	T2N0M0	G3	N.D.	N.D.	7.10	N.D.
15	70	M	T2N0M0	G3	1.72	22.80	24.70	N.D.
19	67	M	T3N0M1	G3	0.87	6.13	23.10	2.03
20	57	F	T3bN0M1	G3	0.78	1.35	0.17	4.60

MMP, matrix metalloproteinase; TIMP, tissue inhibitor of metalloproteinase; ccRCC, clear cell renal carcinoma; N.D., none detectable.
